# Mesenchymal Stromal Cells for Transplant Tolerance

**DOI:** 10.3389/fimmu.2019.01287

**Published:** 2019-06-04

**Authors:** Manuel Alfredo Podestà, Giuseppe Remuzzi, Federica Casiraghi

**Affiliations:** ^1^Department of Molecular Medicine, Istituto di Ricerche Farmacologiche Mario Negri IRCCS, Bergamo, Italy; ^2^Department of Health Sciences, Università degli Studi di Milano, Milan, Italy

**Keywords:** mesenchymal (stromal) stem cells, transplantation, T cells, macrophage, tolerance, B cells

## Abstract

In solid organ transplantation lifelong immunosuppression exposes transplant recipients to life-threatening complications, such as infections and malignancies, and to severe side effects. Cellular therapy with mesenchymal stromal cells (MSC) has recently emerged as a promising strategy to regulate anti-donor immune responses, allowing immunosuppressive drug minimization and tolerance induction. In this review we summarize preclinical data on MSC in solid organ transplant models, focusing on potential mechanisms of action of MSC, including down-regulation of effector T-cell response and activation of regulatory pathways. We will also provide an overview of available data on safety and feasibility of MSC therapy in solid organ transplant patients, highlighting the issues that still need to be addressed before establishing MSC as a safe and effective tolerogenic cell therapy in transplantation.

Transplantation represents the treatment of choice for end-stage solid organ failure. The dissection of the mechanisms regulating the interplay between the host immune system and the transplanted graft have led to the introduction into clinical practice of effective T-cell immunosuppressive agents, which have abated the risk of acute rejection in the peri-transplant period, increasing the 1-year graft survival above 90%. However, current immunosuppressive agents failed to significantly affect the long-term outcome of solid organ transplantation, because these drugs are less effective in preventing chronic allograft rejection (1). Moreover, the lifelong systemic immunosuppression exposes transplant recipients to a significant risk of side effects, infections and malignancy (2, 3).

Since acute T-cell mediated rejection can be successfully managed in most cases, transplant research should focus on the identification of innovative strategies to achieve allograft tolerance, i.e., long-term stable graft function in the absence of immunosuppression. Therefore, the ideal strategy should target not only T cells, which are the main players of alloimmunity, but regulate in a concerted action also B cells, dendritic cells and macrophages, which all contribute to both the acute and chronic alloimmune response.

In this scenario, mesenchymal stromal cells (MSC) seem a very promising cellular therapy in the pursuit of transplantation tolerance induction, allowing minimization or even discontinuation of life-long immunosuppression. Indeed, MSC have a unique capability to inhibit the immune alloresponse at different levels and to dampen the activation of cells of both the adaptive and innate immune systems, reprogramming them into regulatory cells. MSC are a heterogeneous subset of non-hematopoietic cells, currently defined by standard criteria that include the ability to differentiate into tissues of mesodermal lineages *in vitro*, plastic adherence under standard culture conditions, expression of CD73, CD90 and CD105, and lack of CD45, CD34, CD14, or CD11b, CD79α or CD19, and HLA-DR surface antigens (4). Although MSC can be obtained from several adult and fetal tissues (including umbilical cord, Wharton's jelly, amniotic fluid, adipose tissue and dental pulp) (5–9), the bone marrow has been traditionally considered as their main source, and thus bone marrow-derived MSC are the ones best characterized both in humans and animal models.

In this review we will provide a summary of the alloimmune response events that accompany solid organ transplantation, discussing the multiple immune modulatory effects that MSC have demonstrated on both the innate and adaptive arms of the immune system and highlighting their tolerance-inducing properties in preclinical transplant models. We will also provide an overview of available data from clinical trials on MSC infusion in solid organ transplant patients, discussing future perspectives and issues that still need to be addressed.

## The Adaptive Immune System: Terminal Effector of the Alloresponse

The crucial step of the adaptive response is represented by the recognition of donor alloantigens (mostly non-self MHC molecules) by recipient lymphocytes. These cells are activated in the presence of costimulatory molecules, leading to a cascade of events that ultimately precipitates into graft rejection (10).

Transplant rejection results in the generation of cells with either regulatory or effector functions, with the latter prevailing during the alloresponse. Effector functions are mainly mediated by CD8^+^ and CD4^+^ T cells: cytotoxic CD8^+^ T cells, activated by class I MHC-presented antigens, undergo clonal expansion, mature into effector cells and migrate into the graft, where they induce apoptosis and secrete cytotoxic molecules.

Class II MHC-restricted CD4^+^ T lymphocytes differentiate into distinct subsets of helper T cells, depending on the cytokine milieu: IL-12 prompts the differentiation in T_H_1 cells and promotes the secretion of IL-2 and IFNγ, which, respectively, sustain CD8^+^ T-cell proliferation and induce a delayed-type hypersensitivity reaction through macrophage activation (11). T_H_2 polarization is guided by IL-4, which results in the recruitment of graft-damaging eosinophils *via* IL-5 secretion (12). T_H_17 induction is mediated by IL-21, IL-23, IL-6, and TGFβ stimulation. T_H_17 cells play a role in both acute and chronic rejection through the recruitment of neutrophils into the transplanted graft (13). Finally, follicular helper T-cell (T_FH_) differentiation is primarily mediated by IL-12, IL-6, and TGF-β signaling; T_FH_ cells promote B-cell differentiation into antibody-producing plasma cells (14, 15) and are the cells responsible for the development of donor-specific antibodies and antibody-mediated rejection (16, 17).

CD4^+^ T cells may also differentiate into several regulatory subsets, which actively inhibit the alloresponse and therefore constitute an essential part of peripheral tolerance. Regulatory T cells (T_REG_) express the transcription factor FoxP3 and are known to suppress the alloreaction through modulation of antigen presentation, production of anti-inflammatory cytokines, as well as competition and cytolysis of effector T cells (18, 19). T-regulatory type 1 cells (T_R1_) can be induced from naïve T cells upon TCR stimulation in the presence of IL-10; these cells downregulate the alloresponse by producing anti-inflammatory soluble mediators, including IL-10 and TGFβ (20).

B-cell mediated humoral immune responses also play an important role in alloimmunity, which is reflected by the elevated incidence of chronic antibody-mediated rejection in long-term graft recipients. B-cell differentiation into antibody-producing plasma cells and memory B cells after alloantigen uptake depend on cognate interactions with T cells. Conversely, following uptake through their B-cell receptor, B cells can mount alloantigens on class-II MHC and present them to T cells. Specific B-cell subsets have displayed immunoregulatory properties, and have been termed regulatory B cells (B_REG_). B_REG_ are a heterogenous population that promotes the development of T_REG_, suppresses effector T cell differentiation and converts T_H_1 effectors into T_R_1 cells, mainly through secretion of the anti-inflammatory cytokines IL-10 and TGFβ (21, 22).

## The Innate Immune System: Old and Emerging Evidence of A Central Role in Transplant Rejection

Despite the high specificity of the adaptive system for non-self-antigens, the set of allogeneic responses that ultimately lead to transplant rejection derives from a complex interplay with the innate immune system, an interaction that is much more intertwined than originally hypothesized.

Innate immunity relies on the activation of pattern recognition receptors (PRRs), which is induced by evolutionarily preserved molecular motifs from pathogens. However, also self-molecules released in response to cell stress can activate PRRs, triggering the innate immunity. These molecules are termed DAMPs (damage-associated molecular patterns) and are massively released in the context of organ transplantation in response to brain/cardiac death and ischemia-reperfusion injury (23, 24).

The best characterized cellular PRRs are toll-like receptors (TLRs), localized on the outer membrane and on the surface of intracellular vesicles, NOD-like receptors, which are part of the inflammasome complex, and C-type lectin receptors. DAMP-induced signaling through these receptors activates the transcription of specific genes, leading to the secretion of inflammatory cytokines and upregulation of adhesion and costimulatory molecules (25, 26).

DAMP-induced TLR signaling promotes the maturation of dendritic cells (DCs), which represents the pivotal link between innate and adaptive immunity. This process determines a critical switch in the biological functions of DCs in solid organ transplantation: donor DCs migrate from the graft to the lymphoid organs of the recipient and present intact donor MHC-antigen complexes to host T cells (“direct presentation”), inducing the intense activation and rapid proliferation of effector T cells that underlies early rejection episodes. In addition, mature recipient DCs infiltrating the graft can also present processed donor antigens to recipient T cells (“indirect presentation”), providing a weaker but long-lasting stimulation that can eventually result in both acute and chronic rejection (27, 28). In the setting of acute rejection, most of the DCs found in the graft derive from infiltrating monocytes that locally differentiate into antigen-presenting cells (29, 30).

Monocytes can also contribute to transplant rejection by differentiating into macrophages, which frequently represent the majority of graft-infiltrating cells during rejection (31). Similarly to other cell types, macrophages can damage the allograft or have immunoregulatory functions depending on their state of activation. Classically-activated M_1_ macrophages are pro-inflammatory cells that develop upon TLR engagement in the presence of IFNγ, damaging the graft *via* direct cytotoxicity or by inducing a delayed-type hypersensitivity reaction. Notably, these effector functions were shown to be triggered by alloantigens, and cytotoxicity proved to be allospecific after T-helper priming (32, 33). On the other end of the spectrum, alternatively-activated M_2_ macrophages, whose differentiation is guided by IL-4 and IL-13, produce anti-inflammatory cytokines (IL-10, TGFβ), induce regulatory T-cell differentiation and promote tissue repair, mitigating graft damage (34, 35).

## Mechanistic Insights on MSC and Tolerance Induction

MSC infusion has not only shown encouraging results in controlling autoimmunity *in vivo* (36), but has also consistently proven effective in prolonging allograft survival in multiple animal models of solid organ transplantation (37–39) (Table 1). Compelling evidence demonstrated that during the alloresponse these cells can tip the balance between effector and regulatory functions in favor of the latter. MSC reduce the host-vs.-graft response in part through contact-dependent regulation (49, 50) and, most importantly, by secreting soluble factors with paracrine immunomodulatory effects (51, 52). A constantly growing number of soluble mediators have been implicated in MSC-induced functions on the immune system, including indoleamine 2,3-dioxygenase (IDO) (53, 54), nitric oxide (55, 56), TGF-β (49, 57–59), prostaglandin-E_2_ (PGE_2_) (50, 60), heme-oxygenase 1 (HO-1) (61), galectins (62) and HLA-G5 (63). Indeed, MSC-conditioned medium and MSC-derived extracellular vesicles, which both contain these soluble bioproducts, have been increasingly studied as cell-free alternatives to MSC administration (64). In addition, a different approach has been explored by some researchers, who engineered MSC to overexpress and secrete several different soluble mediators, including IDO (65), TGFβ (66), IL-10 (67), and HO-1 (68–70). Preliminary evidence from animal models of solid organ transplantation showed a modest but consistent increase in the pro-tolerogenic activity of genetically modified MSC compared to their standard counterparts (Table 2). Soluble mediators act on multiple cell targets, resulting in dramatic changes in their phenotype and functions.

**Table 1:** MSC in preclinical transplant models.

**Transplant model**	**MSC**			**Outcome**	**Immunological mechanism**	**References**
	**Origin**	**Timing^a^**	**Dose/Route**			
Skin tx in baboons	Donor BM	Day 0	1–2 × 10^7^ /kg	Significant prolongation of graft survival		(40)
Liver tx in rats (LEW in BN rats)	BM from syngeneic, donor or TP (Wistar) rats	Days 0, +1, +2, +3, +8, +12, +16 (7 doses)	2 × 10^6^/dose, IV	Significant prolongation of graft survival irrespective whether MSC were of syngeneic, donor or TP origin	Foxp3^+^ Treg generation	(41)
Kidney tx in mice (C57 in BALB/c)	Donor BM	Day 1	1 × 10^6^, IV	Indefinite graft survival	IDO-dependent Foxp3^+^ Treg generation	(38)
Heart tx in mice (C57 in BALB/c mice)	Donor BM	Day +1	1 × 10^6^, IV	Indefinite graft survival by MSC in combination with low-dose rapamycin	Tolerogenic DC and Foxp3^+^ Treg generation	(39)
Kidney tx in rats (F344 in LEW rats)	TP (SD) BM	Week 11	0.5 × 10^6^, IV	Prevention from chronic renal graft dysfunction and injury (IF/TA)	Anti-inflammatory effects	(42)
Kidney tx in mice (BALB/c in sensitized^b^ C57 mice)	Syngeneic BM	Day−1 or day−7 or double pre-tx infusion (days−7 and−1) or at day +2	0.5 × 10^6^, IV	Significant prolongation of graft survival when MSC were given pre-transplant, acute graft rejection when MSC were given post-transplantation	Foxp3^+^ Treg generation	(43)
Heart tx in rats (Wistar in F344 rats)	Donor BM	Day−7, 0, +1, +2, +3 (5 doses)	2 × 10^6^/dose, IV	Significant prolongation of graft survival	Reduced pro-inflammatory and increased anti-inflammatory cytokine expression	(37)
Heart tx in mice (C57 into BALB/c mice)	Donor adipose tissue	Day−4	1 × 10^6^, IV	Significant prolongation of graft survival by MSC in combination with MMF	Conversion into Foxp3^+^ Tregs (by MMF) of Th17 cells induced by MSC-educated MDSC	(44)
Heart tx in mice (B6C3 in C57 mice)	Syngeneic or donor BM	Days−7 and−1	0.5 × 10^6^, IV (portal vein day−7, tail vein day−1)	Significant prolongation of graft survival with either syngeneic or donor-derived MSC	Foxp3^+^ Treg generation	(45)
Corneal tx in rats (Wistar in LEW rats)	Donor BM	Days−3,−2 and−1 or days 0, 1 and 2	5 × 10^6^, IV	Significant prolongation of graft survival when MSC are given post-transplant either alone or combined with CNI	Foxp3^+^ Treg generation	(46)
Corneal tx in mice (C57 in BALB/c mice)	Human BM	Days−7 and−3	1 × 10^6^, IV	Significant prolongation of graft survival	Conversion of lung monocyte/macrophage toward an immune regulatory phenotype in a TSG-6-depedendent manner	(47)
Corneal tx in rats (DA in sensitized^b^ LEW rats)	TP (Wistar Furth) BM	Days−7 and−1	1 × 10^6^, IV	30-day rejection free in 64% MSC-treated animals compared to 0% in the control group	Induction of PGE_2_/TGFβ-producing and immunosuppressive CD45^+^CD11b^+^B220^+^ lung monocytes and Foxp3+Treg generation	(48)

a *From day of transplant (Day 0)*;

b *Donor-sensitization by donor splenocyte injection prior to transplantation*.

*BM, bone marrow; BN, Brown Norway; CNI, calcineurin inhibitor; DA, Dark Agouti; DC, dendritic cells; IDO, indoleamine 2,3-dioxygenase; IF/TA, interstitial fibrosis/tubular atrophy; IV, intravenous; LEW, Lewis; MMF, mycophenolate mofetil; PGE2, prostaglandin E2; SD, Sprague-Dawley; TGFβ, transforming growth factor β; TP, third-party; TSG-6, tumor necrosis factor-inducible gene 6; Tx, transplant*.

**Table 2:** Preclinical data on genetically-engineered MSC in solid organ transplantation.

**References**	**Type of graft**	**Donor/recipient**	**MSC source**	**Mediator/vector**	**Outcome**	**Immunological effects**
He et al. (65)	Kidney	NZ/Japanese white rabbits	NZ rabbit (BM)	IDO/lentivirus	IDO-MSC prolonged graft survival compared to standard MSC, prevented rejection and induced donor-specific tolerance to skin grafts.	Inhibition of T-cell proliferation, Treg induction and increased levels of tolerance-related cytokines. All effects were greater with IDO-MSC compared to standard MSC.
Tang et al. (66)	Liver	DA/Lewis rats	Lewis rats (BM)	TGFβ/lentivirus	TGFβ-MSC reduced graft rejection and increased survival compared to standard MSC.	Reduced effector T-cell proliferation *in vivo*, Treg induction (but reduction in natural Treg) and increased Treg/T_H_17 ratio in graft-infiltrating cells.
Niu et al. (67)	Liver	DA/Lewis rats	DA rats (BM)	IL-10/lentivirus	IL-10-MSC increased graft survival and reduced the histological rejection activity index compared to standard MSC.	Increased FoxP3 expression in intragraft CD4^+^CD25^+^ T cells, reduced pro-inflammatory and increased anti-inflammatory cytokines.
Wu et al. (68)	Liver	Lewis/BN rats	BN rats (BM)	HO-1/adenovirus	HO-1-MSC increased survival rates and attenuated acute rejection compared to standard MSC.	Increased Treg fraction in splenocytes, induction of an anti-inflammatory cytokine profile.
Wang et al. (69)	Liver (reduced-size)	Lewis/BN rats	BN rats (BM)	HO-1/adenovirus	HO-1-MSC attenuated the rejection activity index compared to standard MSC.	Reduction in mTOR/ERK levels, along with increased autophagy-related proteins (LC3 and Beclin-1).
Yang et al. (70)	Small bowel	BN/Lewis rats	Lewis rats (BM)	HO-1/adenovirus.	HO-1-MSC improved survival rates, clinical manifestations and acute rejection grading compared to standard MSC	Reduction of the pro-inflammatory cytokine milieu, increased Treg fraction in splenocytes.

*BN, Brown Norway; BM, bone marrow; DA, Dark Agouti; HO-1, heme oxygenase-1; IDO, indoleamine 2,3-dioxygenase; IL-10, interleukin-10; MSC, mesenchymal stromal cells; NZ, New-Zealand; TGFβ, transforming growth factor β*.

### T Cells

Autologous and allogeneic MSC regulate the T-cell response to the graft via modulation of alloantigen presentation and through direct, antigen-independent effects on T cell. In their seminal work, Bartholomew et al. demonstrated for the first time that MSC can prevent not only T-cell alloreactivity, but also polyclonal activation and proliferation of baboon T cells induced by mitogens *in vitro* (40). This dose-dependent effect was also confirmed in mice with a series of elegant experiments assessing single-antigen reactivity (71, 72), which showed that MSC inhibition was not limited to antigen-specific T-cell clones, but determined a generalized division arrest anergy in both naïve and memory T cells, eventually resulting in reduced CD8^+^ T-cell proliferation and cytotoxicity. Similar results were obtained with human autologous and allogeneic MSC, which were able to suppress T-cell proliferation due to both antigen-priming and polyclonal activators (73–75). Findings that MSC are able to inhibit memory T cells, including CD8^+^ memory T cells, is of particular relevance, since a high frequency of alloreactive memory T cells before transplantation represents a barrier to tolerance induction (76–78) and threatens allograft survival, especially in the context of T-cell depleting induction therapy (79–82).

MSC can also modify the activity of helper T cells by rewiring their polarization. Addition of MSC to T-cell cultures stimulated by mitogens, primed by allogenic T cells, or under T_H_1-differentiating conditions, suppressed T_H_1-cell proliferation and activation, and promoted a strong T_H_2 polarization along with increased IL-10 production (74, 83, 84). The same effect was observed in a rat model of corneal transplantation, in which MSC injection shifted the balance between T_H_1- and T_H_2-specific cytokines in favor of the latter (46). Moreover, MSC displayed the capacity to inhibit T_H_17 generation by suppressing the transcription factor RORγt and to induce suppressive T_REG_ from differentiated T_H_17 cells, both *in vitro* and *in vivo* (44, 50, 85). Although data regarding MSC effect on T_FH_ cells in solid organ transplantation are currently lacking, MSC were shown to suppress the differentiation and proliferation of cultured T_FH_ cells obtained from healthy human donors (86).

Alongside effector T-cell inhibition, MSC expand several subsets of regulatory cells, making them a unique tool to modulate the alloresponse. In murine models, MSC polarized T cells toward a FoxP3^+^ T_REG_ phenotype both *in vitro* and *in vivo* (41, 45, 83), an effect that was evident also in human T-cell cultures (49, 63) and in transplant recipients undergoing MSC infusion (87). Recently, MSC were also reported to promote IL-10 secretion and to expand the T_R_1 regulatory subset in allogeneic mixed-lymphocyte reactions (MLRs), which mediated immunosuppression *in vitro* through the PGE_2_ and IDO pathways (61, 88).

### B Cells

Different reports have shown that MSC may block B-cell proliferation through cell-cycle arrest into the G0/G1 phase (89, 90). This alteration results in a strong inhibition of proliferation and maturation of B cells into plasmablasts, causing a steep reduction in antibody secretion (91, 92). Of particular interest for the transplantation field, the addition of MSC to standard allogeneic MLRs was shown to inhibit the formation of donor-specific antibodies (92). Interestingly, *in vitro* B-cell proliferation was not suppressed when MSC and B cells were cultured without T cells, suggesting that this effect is at least in part mediated by T-cell help (91). On the other hand, absence of T cells did not interfere with MSC-mediated inhibition of B-cell maturation and with B_REG_ induction (93).

Inflammatory licensing by IFNγ significantly modifies MSC effect on B-cell subsets: MSC cultured in standard conditions were shown to increase the percentage of B_REG_ along with IL-10 production, while MSC exposed to high IFNγ concentrations did not promote B_REG_ expansion, but instead induced a greater inhibition of B-cell proliferation through IDO metabolic effects (94, 95).

Although, to date, there is a lack of studies exploring MSC effect on the B-cell compartment in animal models of solid organ transplantation, MSC infusion prevented the formation of circulating donor-specific antibodies in rats undergoing allogeneic kidney transplantation, suggesting that these cells are able to modulate humoral responses *in vivo* (42). Moreover, long term immunophenotyping of kidney allograft recipients that received MSC infusions revealed increased naïve and CD24^HI^CD38^HI^ (“transitional”) B-cell subsets, a phenotypic signature that was associated with spontaneous operational tolerance (87, 96). Additional characterization will be needed to clarify whether MSC may have increased the frequency of IL-10 producing B cells (i.e., those associated with B_REG_ phenotype and function), especially in the “transitional” B-cell fraction.

### Dendritic Cells

MSC modulate DC phenotype and function at multiple levels, inducing pro-tolerogenic functions. Exposure to mouse MSC interferes with DC maturation, downregulating class II MHC and the costimulatory molecules CD40 and CD86 (97, 98). Moreover, MSC also impair DC homing to secondary lymphoid organs by reducing the expression of CCR7 and CD49dβ1 (99).

These effects have direct consequences on alloantigen presentation by both donor and recipient DCs: since MLR reactivity mostly depends on direct antigen presentation, the inhibition of proliferation observed in these cultures after MSC addition suggests an inhibitory effect on “donor” DCs (97). This was confirmed by the hypo-responsiveness displayed by human allogeneic T-cell responders to MSC-primed DCs compared to mature DCs (100–102). *In vitro* experiments with ovalbumin-specific TCR-transgenic mouse T cells demonstrated that MSC can also exert regulatory effects on ovalbumin-pulsed “recipient” DCs, leading to defective indirect alloantigen presentation to CD4^+^ T cells and reduced cross-presentation of allopeptides to CD8^+^ T cells (98, 99).

DC maturation block and inefficient antigen presentation, coupled with lack of co-stimulation, induces a regulatory phenotype from conventional T cells, thereby increasing the relative abundancy of T_REG_ compared to effector T cells and inhibiting the alloresponse. In an allogeneic kidney transplant model, MSC infusion was associated with high frequency of immature, tolerogenic DCs, along with impaired donor-specific T-cell proliferation and enrichment of suppressive T_REG_ in secondary lymphoid organs and into the graft (38). Similar results had been previously observed in mice receiving a cardiac allograft, in which tolerance was achieved by combining MSC infusion with low-dose rapamycin (39).

### Macrophages

Several models have shown that MSC can increase macrophage proliferation and migration, while inducing a pro-tolerogenic polarization shift at the same time: MSC-reprogrammed M_2_ macrophages show reduced TNFα, IFNγ, and IL-12 secretion and increased IL-10 production, which inhibit effector T-cell responses and promote T_REG_ proliferation (59, 103–105).

Several soluble mediators secreted by MSC have been implicated in macrophage reprogramming, including IDO and PGE_2_ (105, 106). Recent reports, however, have shown that also phagocytosis of apoptotic MSC or MSC microparticles by macrophages is sufficient to induce an M_2_ polarization shift, partly through IDO upregulation in these phagocytes (107–109). Moreover, macrophage uptake of the cell debris deriving from MSC-induced T-cell apoptosis also induced TGFβ secretion, which facilitated T_REG_ expansion (110).

*In vivo*, MSC infusion before allogeneic corneal transplantation redirected macrophages toward an M_2_ phenotype, which conferred protection against allograft rejection (47). In this model, adoptive transfer and selective depletion experiments suggested that the monocyte lineage was responsible for tolerance induction (47). In pre-sensitized rats, allogeneic MSC administration before corneal transplant significantly increased rejection-free survival, which was associated with an early rise in alternatively-activated macrophages, followed by an increase in T_REG_ at later time points (48).

## Dual Function of MSC and Crosstalk With the Microenvironment

Although MSC can act as potent immunomodulators of inflammation during the alloresponse, in resting conditions they mainly display homeostatic properties, supporting cells residing in their niche (111). Moreover, MSC can also acquire a completely opposite function, releasing pro-inflammatory cytokines and even acting as antigen-presenting cells following class II MHC upregulation (112–114). The acquisition of either of these specific functions strictly depends on the microenvironment MSC encounter: to become immunosuppressive, MSC need to “licensed” by a series of inflammatory stimuli that commonly occur in the setting of solid organ transplantation, deriving from both the innate and adaptive immune response.

Cytokines are the best studied mediators involved in MSC licensing: binding of IFNγ (chiefly secreted by T-cells after T_H1_ polarization) to its specific receptors on MSC represents a critical step of this process (115, 116). Other cytokines, including the macrophage-derived TNFα, IL-1α and IL-1β, have been shown to potentiate the licensing effect of IFNγ (55). Overall, this stimulation results in the production of chemokines (such as CXCR3 and CCR5) and in the induction of IDO, which are respectively necessary for T-cell recruitment and inhibition. However, below-threshold IFNγ and TNFα concentrations have been proven insufficient to induce IDO, therefore precluding the suppression of recruited T-cells and paradoxically increasing the inflammatory response (117).

MSC functional destiny is also influenced by the dynamics of DAMP-associated signaling that accompanies tissue injury. The presence of a wide array of TLRs has been confirmed on the surface of both human and mouse MSC (118–120), with variable expression influenced by environmental cues, including hypoxia and inflammatory cytokines (114, 121). TLR stimulation promotes MSC migration to the site of inflammation and enhances their survival (122, 123), but conflicting data have been reported regarding a DAMP-mediated licensing effect through TLR3 and TLR4 stimulation (124, 125). Recent evidence suggests that MSC can be differentially activated based on the type of TLR triggered: transient TLR4 engagement by low lipopolysaccharide concentrations polarizes MSC toward a proinflammatory phenotype, stimulating the secretion of IL-6 and IL-8, recruiting neutrophils and inducing T-cell activation in co-culture experiments (126, 127). On the other hand, sub-maximal triggering of TLR3 guides the differentiation into immunomodulating MSC, which upregulate IDO, secrete IL-10, CXCL10 and CCL5, and induce T-cell suppression *in vitro* (126).

In addition to its multiple pro-inflammatory effects, activation of the complement cascade also has direct effects on MSC activity. MSC are resistant to complement-mediated lysis due to expression and secretion of negative regulators (128, 129) but, at the same time, ligation of complement anaphylatoxins to their surface receptors enhances MSC resistance to oxidative stress and apoptosis (130). Although a direct licensing effect of complement has not been described, C3a and C5a act as potent chemotactic agents for MSC and recruit them at the site of inflammation, where functional polarization takes place (131, 132).

## Timing as a Key Factor for MSC-Induced Tolerance

MSC survival is limited both in culture systems and following *in vivo* administration (45, 133). This peculiar feature seems antithetic to the development of long-term tolerance after a single MSC infusion, unless one takes into account that these cells may determine a multi-level protolerogenic polarization within a limited time frame. Therefore, the reduced lifespan of MSC raises by itself the issue of optimal infusion timing in allograft recipients. This concern was further corroborated by the first evidence that the timing of MSC infusion dictates their pro-tolerogenic effect: in an allogeneic rat model of heart transplantation, donor MSC infused 4 days before the transplant induced acceptance of the graft, as opposed to infusions on the same day or after 3 days from the procedure (134).

Building upon data indicating that systemically infused MSC tend to migrate to damaged tissues (such as those exposed to ischemia/reperfusion injury) (135–137), we hypothesized that timing of infusion may impact on MSC localization, and confirmed that administration of MSC after transplantation results in migration of these cells inside the graft, rather than toward secondary lymphoid organs (43). In addition, the site at which MSC are recruited tightly correlates with their immunomodulatory properties: indeed, migration of MSC into kidney allografts following post-transplant infusion was associated with a proinflammatory phenotype and resulted in neutrophilic infiltration, complement deposition and acute kidney injury, both in animal models and humans (43, 87). The events leading to this polarization are still ill-defined, but the inflammatory microenvironment and the massive release of DAMPs are likely key factors. On the other hand, administration of MSC before renal transplantation led to localization of these cells into secondary lymphoid organs, where they promoted the formation of a pro-tolerogenic environment and prolonged graft survival (43).

Localization by itself, however, does not fully explain the opposing characteristics that MSC may acquire in the setting of solid organ transplantation. We recently showed that experimental inhibition of C3a and C5a receptors on MSC infused after kidney transplantation prevents intra-graft migration and allows homing of these cells to secondary lymphoid organs. However, despite correct localization and reduction of donor-specific T-cell alloreactivity, MSC failed to induce T_REG_ generation in this model and resulted only in limited prolongation of graft survival (132).

These experiments confirmed previous evidence suggesting that the effect of MSC on alloreactive T cells also depends on the degree of maturity these T cells display. Indeed, when MSC were added to MLRs after the primary stimulation step had already taken place, CD8^+^ T-cell mediated cytotoxicity was unhindered, suggesting that a complete polarization favors the escape from MSC-mediated immunoregulation (138). Similarly, the *in vitro* regulatory effect of MSC on T_H_1/T_H_17 proliferation, activation and differentiation was progressively lost when MSC were added with increasing delay from the start of the polarization process (83).

Consistent with effects observed in mice, MSC added at later time points *in vitro* were also unable to convert terminally differentiated conventional T cells into T_REG_ (83, 139). Among mediators involved in T_REG_ induction, the trophic cytokine IL-2 seems to be critical for the whole process to occur (140, 141): since neither MSC nor T_REG_ secrete IL-2, the concentration of this cytokine relies on conventional T-cell production. Studies on the kinetics of IL-2 secretion during effector T-cell differentiation elucidated how terminal effectors progressively lose their ability to secrete this cytokine (142), resulting in a steep decline in IL-2 concentrations after 2 days in a conventional MLR (143). Thus, it is tempting to speculate that MSC may fail to induce T_REG_ from terminally differentiated conventional T cells due to low IL-2 concentrations, but other concomitant factors are likely to play a role. For instance, timing seems also involved in MSC-mediated induction of an immature, tolerogenic DC phenotype: addition of lipopolysaccharide to MSC-DC co-cultures, mimicking the DAMP-induced TLR triggering that accompanies graft transplantation, reduced the inhibitory effect of MSC on DC maturation (144, 145). As previously described, the acquisition of a mature DC phenotype prevents T_REG_ expansion, thus providing an additional plausible explanation of the reduced immunomodulating ability of MSC infused post-transplantation.

Failure to expand T_REG_ has been frequently associated with graft rejection in animal models (38, 43), suggesting a central role for suppression in MSC-induced tolerance. Thus, infusion protocols aimed at expanding the T_REG_ pool should have the highest potential of promoting a protolerogenic environment. Pre-transplant MSC administration has the best chances to achieve this aim, even though T_REG_ expansion in the absence of antigenic pressure from the graft produces a broad repertoire, which includes both donor-specific and non-alloreactive regulatory T cells.

**Figure 1:**
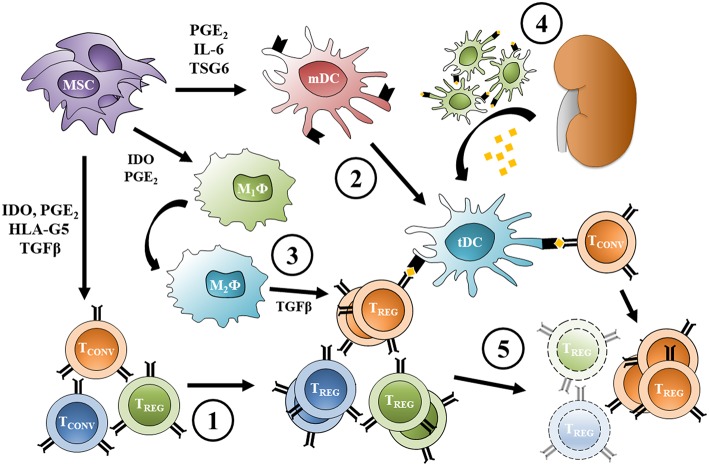
Expansion of donor-specific T_REG_ following pre-transplant MSC infusion. (1) MSC infused before transplant induce T_REG_ from conventional T cells and promote a polyclonal, antigen-independent expansion; at the same time, (2) MSC inhibit dendritic cell maturation increasing the frequency of protolerogenic DCs, and (3) reprogram macrophages toward an M_2_ phenotype. (4) Allogeneic transplantation causes migration of donor DCs from the graft to lymphoid organs and provides alloantigen for recipient DC uptake (other antigen presenting cells can act the same way). (5) Direct and indirect antigen presentation provide the survival stimuli necessary for donor-specific T_REG_ proliferation, while the non-alloreactive T_REG_ pool is progressively lost over time. Protolerogenic DCs can also induce T_REG_ from conventional alloreactive T cells, while alternatively-activated macrophage TGFβ secretion promotes T_REG_ expansion. MSC, mesenchymal stromal cell; T_CONV_, conventional T cell; T_REG_, regulatory T cell; mDC, mature dendritic cell; tDC, tolerogenic dendritic cell; M_1_ϕ, classically-activated macrophage; M_2_ϕ, alternatively-activated macrophage; PGE2, prostaglandin-E_2_; IDO, indoleamine 2,3-dioxygenase; TGFβ, transforming growth factor β; HLA-G5, human leukocyte antigen G5; IL-6, interleukin 6; TSG6, tumor necrosis factor-inducible gene 6 protein.

Indeed, antigen-pressure is not necessary to induce suppressive T_REG_ from conventional T cells in MSC co-culture (146). However, FoxP3 expression in these cells is not stable, suggesting that polyclonal T_REG_ induction without alloantigen presentation does not provide the required survival and proliferation stimuli for a stable long-term expansion (146). *In vivo* administration of MSC to healthy, non-transplanted mice expands the T_REG_ pool (43), and adoptive transfer of splenocytes from these mice to syngeneic transplant recipients induces tolerance to the allograft (45), consistent with the notion that polyclonally-expanded T_REG_ can modulate the alloresponse. However, after graft tolerance has been established, T_REG_-mediated suppression becomes donor-specific, without affecting the response to third-party alloantigen presentation *in vivo* (45). These findings suggest that MSC infused before the transplant and localizing to secondary lymphoid organs induce a polyclonal, antigen-independent expansion of T_REG_. Antigenic pressure from the graft then provides the required survival and expansion stimuli to donor-specific T_REG_, while non-alloreactive T_REG_ clones are progressively lost over time (Figure 1).

## MSC and Solid Organ Transplantation: Open Issues for Clinical Translation

Cellular therapy with MSC has been tested so far in phase 1 clinical studies in kidney, liver and lung transplantation (Table 3) using either autologous bone marrow- (BM-)derived (87, 147–151), allogeneic BM-derived (152, 155–158) or umbilical cord-derived MSC (154). These studies have indicated the safety, feasibility and tolerability of the procedure in all settings, including the intravenous injection of MSC in lung transplant recipients with moderate obstructive chronic lung allograft rejection (158). All MSC-treated patients showed good graft function over the 1–2 years-follow-up.

**Table 3:** Summary of clinical trials assessing MSC infusion in the setting of renal, liver and pulmonary transplantation.

**References**	**Number of patients**	**Source of MSC**	**Timing**	**MSC dose**	**Clinical outcome**	**Immunological outcomes**
**KIDNEY TRANSPLANTATION**						
Tan et al. (147)	105 MSC 51 controls	Autologous BM	Day 0, day +14	1–2· × 10^6^/Kg	Safety and feasibility MSC can replace induction therapy with basiliximab and allow the reduction of CNI dose	N/A
Perico et al. (87, 148, 149)	5	Autologous BM	Day +7 (*n* = 2) Day−1 (*n* = 3)	1–2· × 10^6^/Kg	Tolerability and feasibility Safetyproviding that MSC are infused at day−1	High ratio Treg/memory CD8^+^ T cells, donor-specific CD8^+^ T cell unresponsiveness, safe IS withdrawal in 1 patient
Reinders et al. (150)	6	Autologous BM	Month 6–10 post-transplant (2 infusions)	1· × 10^6^/Kg	Safety, tolerability and feasibility Resolution of IF/TA in 2 patients Development of opportunistic infections in 3 patients	Decreased proliferation and cytokine production in response to donor cells
Mudrabettu et al. (151)	4	Autologous BM	Day−1 and day +30	0.2–0.3 (low dose) or 2.1–2.8 (high dose) × ·10^6^/Kg	Safety, tolerability and feasibility	Trend of increased peripheral Treg percentages, reduced polyclonal CD4^+^ T cell proliferation
Peng et al. (152), Pan et al. (153)	16	Allogeneic BM	Day 0, day +30	5· × 10^6^ intragraft, then 2· × 10^6^/Kg	Safety, tolerability and feasibility MSC allow 50% reduction of CNI dose	No significant changes in Treg percentages compared to controls or to basal values
Sun et al. (154)	21 MSC 21 controls	Umbilical cord	Day−1 and day 0	2· × 10^6^/Kg pre-Tx, 5·× 10^6^ intragraft	Safety, tolerability and feasibility	N/A
Erpicum et al. (155)	10 MSC 10 controls	Allogeneic BM	Day 3 ± 2	1.5–3.0· × 10^6^/Kg	Safety, tolerability and feasibility Potential long-term immunization against MSC	Increased frequency of Treg
**LIVER TRANSPLANTATION**						
Soeder et al. (156)	1	Allogeneic BM	Day 0, day +2	150· × 10^6^	Clinically diagnosed acute rejection at day 6 and biopsy proven acute rejection at day 219	Increased frequency of Tregs and reduced expression of HLA-DR on CD14^+^ monocytes
Detry et al. (157)	10	Allogeneic BM	Day 3 ± 2	1.5–3.0· × 10^6^/Kg	Safety, tolerability and feasibility Failure to withdraw IS	No significant changes in Treg counts or phenotype compared to controls
**LUNG TRANSPLANTATION**						
Keller et al. (158)	9	Allogeneic BM	–	1, 2 or 4· × 10^6^/Kg (three groups)	Safety, tolerability and feasibility	N/A

*BM, bone marrow; CNI, calcineurin inhibitor; IF/TA, interstitial fibrosis/tubular atrophy; IS, immunosuppression; MSC, mesenchymal stromal cells; N/A, not available*.

Even though an initial study in kidney transplantation reported a high incidence of opportunistic infections in MSC-treated kidney transplant recipients (150), subsequent studies allayed this concern. In kidney and liver transplant recipients MSC infusion was associated with a reduced (147) or unchanged (87, 155) incidence of opportunistic infections in the first 1–2 years post-transplant compared to controls. In our kidney transplant patients given autologous BM-derived MSC we did not observe increased susceptibility to infection or neoplasm even in the long term (5–7-years follow-up) (87).

The hypothetical increased risk of malignancy following MSC administration constitutes an additional matter of debate (159). This concern originally stemmed from the observation that murine MSC may undergo spontaneous malignant transformation during *ex vivo* cultures (160). However, this feature of murine MSC is not shared by their human counterparts, which are not prone to maldifferentiation even after long-term *in vitro* expansion (159, 161). Despite the lack of malignant potential, human MSC may still theoretically promote the growth of pre-existing undiagnosed tumors in transplant recipients. Preclinical evidence in this context is not univocal, with MSC effects varying from neoplastic facilitation to anti-tumoral activity (159, 162–164). Even though studies carried out so far did not detect any association between MSC infusion and malignancy (165), long-term surveillance systems need to be established in order to gauge a precise estimate of adverse events related to MSC administration.

A major concern regarding the use of third-party MSC therapy refers to possible recipient sensitization due to development of antibodies against allogeneic MSC, a phenomenon demonstrated in animal models (166, 167). This fundamental issue, however, has been considered only by one recent study in kidney transplantation (155): ten kidney transplant recipients from deceased donor were given third-party BM-derived MSC on day 3 ± 2 post-transplant. During the 12 months-follow up, four MSC-treated patients developed *de novo* antibodies against MSC or shared kidney donor-MSC HLA, albeit at very low levels and with only one patient showing *de novo* MSC-donor-specific antibodies with MFI > 1,500. The clinical significance of these alloantibodies is uncertain and further studies with longer follow-up are needed to definitely address the issue of possible sensitization after allogeneic MSC injection.

The available studies in kidney transplantation also provide some evidence of potential efficacy of the MSC therapy in enabling minimization of induction (147) and maintenance (152, 153) immunosuppressive drugs, in inducing a pro-tolerogenic environment (87, 148, 149) and in repairing chronic allograft damage (150).

In a large clinical trial conducted in China, autologous BM-MSC infusions (at reperfusion and 2 weeks later, 1–2 × 10^6^/Kg each) could safely replace induction therapy with the anti-IL-2R antibody basiliximab and enable 20% CNI dose reduction in living donor kidney transplantation (147). In a subsequent study from China, allogeneic BM-derived MSC allowed a 50% reduction of tacrolimus dose used as maintenance therapy. Patients given MSC showed graft survival, incidence of acute rejection and graft function similar to the control patients receiving a full tacrolimus dose (153).

We are currently conducting clinical studies of MSC therapy in living (NCT00752479 and NCT02012153) (87, 148, 149) and deceased donor kidney (NCT 02565459) and liver (NCT02260375) transplantation. In our experience with autologous BM-derived MSC infusion in living donor kidney transplant recipients we showed that timing of cell infusion was crucial in establishing the eventual effect of MSC. MSC infusion at day 7 after transplantation on top of induction therapy with low dose thymoglobulin and basiliximab, resulted in transient renal insufficiency in the first two patients (148), likely caused by MSC migration toward the inflamed graft with the consequent activation of their pro-inflammatory phenotype. We therefore modified the protocol and the subsequent two patients were given infusion of autologous MSC the day before kidney transplantation (149). In this setting, we removed basiliximab from the induction therapy to avoid any deleterious effect of the anti-IL-2R antibody on T_REG_ possibly expanded by MSC. MSC infusion was no longer associated with acute renal insufficiency (149) but the basiliximab-free regimen exposed patients to an increased risk of acute rejection. The protocol was therefore further modified by re-introducing basiliximab induction therapy in the setting of pre-transplant (day−1) MSC infusion. Two additional patients have been treated so far with this protocol and results of 1 year follow up of the first patient have been recently published (87). The first four patients are now at 5–7-years follow-up with stable graft function and no major side effect (87). In these patients we performed extensive longitudinal immunological studies and were able to document a long lasting increase in the ratio between T_REG_ and memory CD8^+^ T cells, along with a persistent reduction of donor-specific CD8^+^ T-cell cytotoxicity *ex-vivo*. These parameters were particularly evident in two patients and were associated with high levels of circulating naïve and transitional B cells, parameters proposed as the B-cell signature of spontaneous and induced tolerance to kidney transplantation (96). In one of these two patients, who was also showing normal histology at 1-year protocol biopsy and no evidence of *de novo* donor-specific antibodies, we attempted immunosuppressive drug withdrawal. Cyclosporin was successfully withdrawn, mycophenolate mofetil was gradually tapered, and the patient is currently free from immunosuppression (Perico et al. manuscript submitted).

Analogously to our approach, the group of Detry et al. attempted immunosuppression withdrawal in liver transplant patients given allogeneic MSC infusion, despite the lack of laboratory evidence of a pro-tolerogenic environment (unchanged percentages of total T_REG_ or their naïve, resting, activated and proliferating subsets) in MSC-treated patients compared to controls. Immunosuppression withdrawal, attempted in nine MSC recipients, was unsuccessful and immunosuppressive drug discontinuation was not achieved (157). Ineffective MSC-mediated immunomodulation in these patients could be due, at least in part, to the absence of a peri-transplant T-cell depleting induction therapy, which would have likely enabled MSC to further expand T_REG_ while restraining memory T cells at the same time.

Overall, the available studies indicate the safety and feasibility of MSC infusion of both autologous and allogeneic source in solid organ transplant recipients. Some suggestions of possible therapeutic efficacy derived from the evidence that MSC allowed minimization of immunosuppression and could promote a T_REG_-mediated pro-tolerogenic environment.

Many open issues such as source, number of infusions, extent of *in vitro* expansion and concomitant immunosuppression regimens still need to be addressed before MSC can become a standard treatment protocol for transplant recipients. In particular, large variations in the dose of MSC infusions are a common occurrence in clinical trials, since the choice is empirical. No direct comparison of MSC doses has been performed in solid organ transplantation so far, but a clinical trial on graft-vs.-host disease showed that amounts ranging from 0.4 to 9.0 × ·10^6^/Kg were not only safe and well tolerated, but also had a similar immunomodulatory effect (168). On the other hand, lack of clinical trial uniformity regarding timing of MSC infusion likely reflects organizational issues rather than insufficient scientific evidence. In light of preclinical and clinical data available, MSC infusion should be always programmed before the transplantation procedure in kidney allograft recipients, in order to avoid potential graft damage resulting from infiltrating proinflammatory MSC. Similarly, the lack of tolerance induction in liver transplant recipients suggests that post-transplant MSC infusion does not efficiently modulate the alloresponse, an effect that might be obtained instead by a pre-transplant administration schedule.

Another fundamental question remains to be addressed, i.e., the final aim of using MSC cellular therapy in solid organ transplantation. We think that MSC should not be adopted to prevent acute allograft rejection, since this phenomenon is well controlled by conventional, less expensive and effective immunosuppressive drugs. Instead, MSC should be harnessed for complementing the tolerogenic potential of induction therapies, which should still be used for the prevention of acute graft rejection, allowing MSC to create a pro-tolerogenic environment at later stages. To this purpose, future efforts should focus on a deeper understanding of mechanism of action of MSC and their interplay with immune cell subsets, with the aim to identify biomarkers of response to MSC therapy that may allow to select patients amenable to safe immunosuppressive drug withdrawal.

## Author Contributions

MP and FC searched the literature and wrote the manuscript. GR critically revised the manuscript.

### Conflict of Interest Statement

The authors declare that the research was conducted in the absence of any commercial or financial relationships that could be construed as a potential conflict of interest.

## Abbreviations

APC: antigen presenting cell
B_REG_: regulatory B cell
DAMP: damage-associated molecular pattern
DC: dendritic cell
HO-1: heme-oxygenase 1
IDO: indoleamine 2,3-dioxygenase
IFNγ: interferon gamma
MLR: mixed-lymphocyte reaction
MSC: mesenchymal stromal cell
NK: natural killer
PGE_2_: prostaglandin-E_2_
PRR: pattern recognition receptor
T_FH_: follicular helper T-cell
T_FR_: T-follicular regulatory cell
TGFβ: transforming growth factor β
T_H_: helper T cell
TLR: toll-like receptor
TNFα: tumor necrosis factor α
T_R1_: T-regulatory type 1 cell
T_REG_: regulatory T cell.
